# Occurrence of Chicken Infectious Anemia Virus in Industrial and Backyard Tunisian Broilers: Preliminary Results

**DOI:** 10.3390/ani12010062

**Published:** 2021-12-28

**Authors:** Antonietta Di Francesco, Giulia Quaglia, Daniela Salvatore, Sonia Sakhria, Elena Catelli, Ghaith Bessoussa, Khaled Kaboudi, Noureddine Ben Chehida, Caterina Lupini

**Affiliations:** 1 Department of Veterinary Medical Sciences, University of Bologna, Ozzano dell’Emilia, 40064 Bologna, Italy; giulia.quaglia2@unibo.it (G.Q.); daniela.salvatore2@unibo.it (D.S.); elena.catelli@unibo.it (E.C.); caterina.lupini@unibo.it (C.L.); 2 Institute of Veterinary Research of Tunisia, University of Tunis El Manar, Tunis 1006, Tunisia; sakhrias@yahoo.fr (S.S.); nbenchehida@yahoo.fr (N.B.C.); 3 Commissariat Régional au Développement Agricole, Ben Arous 2063, Tunisia; ghaithbessoussa@hotmail.fr; 4 National School of Veterinary Medicine of Sidi Thabet, University of Manouba, Sidi Thabet 2020, Tunisia; khaled.kaboudi@enmv.uma.tn

**Keywords:** Gyrovirus, chicken infectious anaemia virus, chickens, PCR, Tunisia

## Abstract

**Simple Summary:**

Chicken infectious anemia virus (CIAV) is an immunosuppressive pathogen of chickens. In the present study, CIAV DNA was detected in a rural broiler farm in Tunisia, whereas the industrial farms sampled were negative. These results underline the importance of constant CIAV surveillance in backyard chicken Tunisian production, considering the potential role of backyard chickens as a reservoir of avian pathogens for intensive breeding.

**Abstract:**

Chicken infectious anemia virus (CIAV) is an economically important and widely distributed immunosuppressive agent in chickens. This study performed an epidemiological investigation on CIAV circulation in 195 Tunisian broilers, belonging to 13 lots from five industrial farms and in one rural farm. Fifteen animals were detected positive by a VP1 nested PCR. The amplicons were molecularly characterised by complete genome sequencing. All positive samples obtained in this study were from the rural farm, whereas the industrial farms sampled were negative. Nucleotide and amino acid sequence analyses showed a high degree of similarity among the sequences obtained, suggesting the circulation of a single CIAV strain in the positive lot. Phylogenetic analysis based on the CIAV VP1 nucleotide sequence and/or the complete genome showed that the sequences obtained in this study clustered with CIAV strains previously detected in Tunisia, Italy and Egypt, belonging to genogroup II. Our results highlight the need for constant CIAV surveillance in backyard chicken production.

## 1. Introduction

Chicken infectious anemia virus (CIAV) is a small, non-enveloped 25-nm icosahedral virus currently belonging to the genus Gyrovirus, family *Anelloviridae* [1,2]. The CIAV genome consists of a molecule of circular single negative-stranded DNA of 2.3 kb [3] that has three partially overlapping open reading frames coding for proteins of 52 (viral protein 1 or VP1), 24 (viral protein 2 or VP2) and 14 (viral protein 3 or VP3) kDa, respectively [4]. Among these viral proteins, the major capsid protein VP1 is associated with viral replication, cell infection ability and virulence [5]. The non-structural VP2 is a dual-specific protein phosphatase that acts as a scaffolding protein to assist in the correct conformation of VP1 [6]. Both VP1 and VP2 can induce neutralising antibodies [7]. Another non-structural protein, VP3, termed apoptin, induces apoptosis in chicken lymphoblastoid T and myeloid cells [8].

Unlike the highly conserved VP2 and VP3 gene sequences, VP1 shows a hypervariable region spanning from residues 139 to 151. Certain amino acid exchanges could influence the rate of virus replication and the spread of CIAV strains in the cell culture [9]. Therefore, VP1 is generally used for genetic characterisation and molecular studies of CIAV. Phylogenetic analysis has been performed on the CIAV VP1 gene or CIAV complete genome, allowing to recognise different genogroups [10,11,12,13,14], although an unambiguous classification has not yet been adopted.

The CIAV genome undergoes recombination events, which may cause the emergence of novel genotypes, altering the epidemiologic picture of this virus and the effectiveness of the current vaccines [13,15,16,17]. Thus, molecular monitoring of the CIAV strains circulating throughout the world could be useful for new approaches in vaccination design [18].

The targets of CIAV are the hemocytoblast and T-lymphocyte precursor cells. Destruction of erythroid progenitors in bone marrow results in severe anaemia, as well as the depletion of granulocytes and thrombocytes. CIAV-induced immunosuppression can result from the destruction of T-lymphocyte precursor cells, with consequent effects on the susceptibility to secondary infectious agents and sub-optimal antibody responses. The chicken B cells and their precursors are not susceptible to CIAV infection [19]; CIAV also infects non-lymphoid cells, supporting its persistence in the reproductive tract and vertical transmission [20]. In addition, CIAV has been reported to be transmitted horizontally in adult chickens by oral or respiratory route [21].

Chickens of all age groups are susceptible to CIAV infection, showing different reactions. Clinical forms occur when susceptible chickens are vertically infected very early, within the first two weeks of age. Clinical signs consist of weakness, depression, anorexia, stunting and runting, as well as increased mortality associated with lymphoid atrophy, bone marrow aplasia and intramuscular haemorrhages [19]. The infection of chickens older than two weeks of age usually proceeds in an asymptomatic form, with indirect evidence of immunosuppression, such as opportunistic secondary infections, increased pathogenicity of several agents such as Marek’s Disease Virus, Reovirus, Infectious Bursal Disease Virus, Avian orthoavulavirus 1 (Newcastle Disease Virus) and inadequate immune response to vaccinations [22].

Due to its severe immunosuppressive potential and the ability to predispose to several secondary infections, CIAV is considered an emerging pathogen potentially responsible for severe economic damage to the poultry industry. The economic losses attributable to CIAV consist mainly of scarce growth, increased mortality, carcass condemnations and antibiotic expenses to control secondary bacterial infections [16].

Breeder vaccination programmes combined with good poultry health and standard management practice represent the main strategy for controlling the spread of CIAV. Live attenuated vaccines are currently used in the poultry industry.

The role of CIAV in the African poultry context has been investigated in Nigeria, Egypt, the Central African Republic, Cameroon, and Libya by serological [23,24,25,26,27,28] or molecular analyses [10,24,26,27,29,30,31]. Seroprevalence values ranging from 37 to 89%, depending on the age of the chicken, have been detected [32]. Most of the serological studies involved commercial chickens. However, Oluwayelu and Todd [24] and Gerish et al. [28] detected specific antibodies against CIAV in 100 of 151 (66.2%) and 69 of 96 (71.8%) backyard chicken sera from Nigeria and Libya, respectively. With regard to the molecular investigations, they were mostly used for additional studies on suspected cases rather than for epidemiological investigations, except for the molecular epidemiological study performed by Ducatez et al. [10] in Nigeria, reporting 30 complete CIAV VP1 sequences from 14/23 different flocks and 8/8 poultry farms.

In Tunisia, breeder CIAV vaccination was implemented a few years ago with live attenuated vaccines during the rearing period of pullets, 6–8 weeks before transfer to the reproduction site. So far, the vaccination programme has been involving only a few breeder farms. Chicken infectious anemia is sometimes described by practitioners in industrial broiler flocks issued usually from unvaccinated breeders. However, the precise prevalence of the disease is unknown.

The aim of this study was to molecularly evaluate CIAV circulation in Tunisian broiler farms and to phylogenetically characterise the detected strains.

## 2. Materials and Methods

### 2.1. Sampling

**Figure 1:**
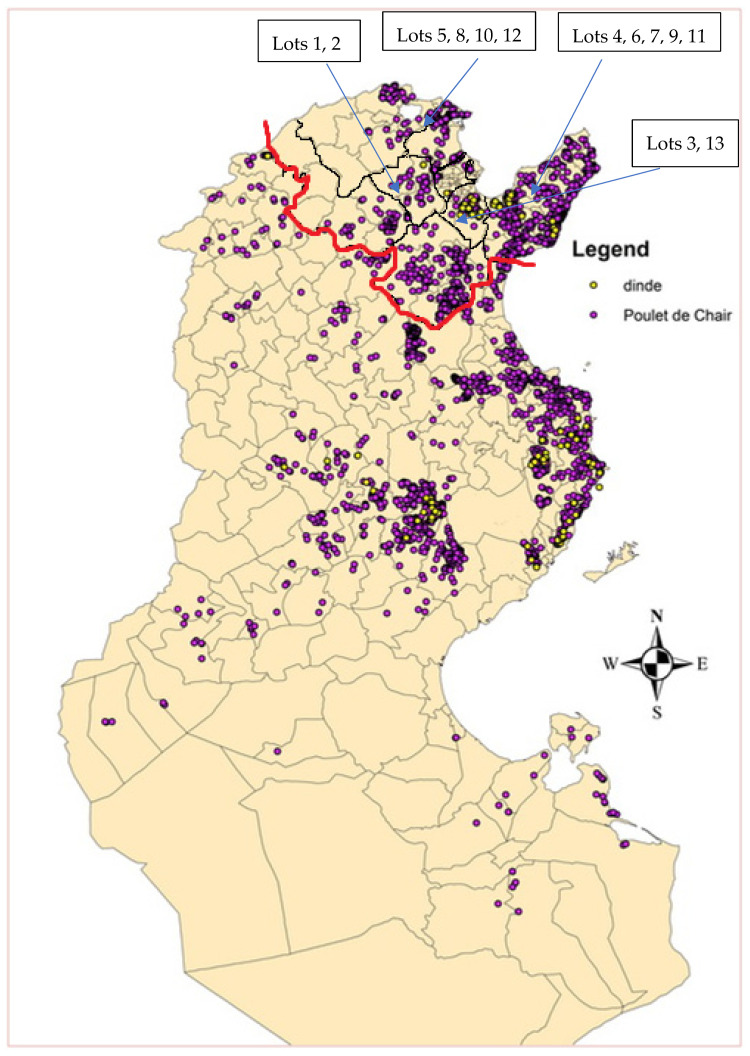
A map of Tunisia showing the location of broiler and turkey farms. Chickens sampled in this study came from farms located in the area bounded by the red line. Source: census report of poultry farms in Tunisia (2015–2016) by General Direction of Veterinary Services (DGSV).

During the period of February to March 2019, individual cloacal swabs were collected from 195 apparently healthy broiler chickens at two slaughterhouses in the governorate of Ben Arous (Grand Tunis, Tunisia). Chickens belonged to 13 lots from six farms (A–F), located in five governorates (Ben Arous, Bizerte, Béja, Zaghouan and Nabeul), in a perimeter of 60 km (Figure 1). All farms were industrial, except for one rural chicken farm. Each lot consisted of 15 animals randomly selected. The slaughter age of the chickens was 35–42 days for industrial chickens and 68 days for rural chickens. Samples were termed as follows: CIAV/Country of origin (Tunisia = TN)/Number of lot-number of the sample.

### 2.2. DNA Extraction

Total DNA was individually extracted from each sample using the QIAamp DNA mini kit (Qiagen, Hilden, Germany) following the manufacturer’s instructions. One extraction control was also included in each PCR analysis, consisting of kit reagents only.

### 2.3. Molecular Screening and Total Genome Amplification

Extracted DNAs were first subjected to a nested VP1 PCR protocol for CIAV screening, employing a primer set previously described [33]. Subsequently, the full CIAV genome from VP1 positive samples was amplified by three overlapping PCRs [13]. The PCR products were separated on agarose gel (2%), stained with MIDORI Green Advance and visualised under ultraviolet light after an electrophoretic run at 100 V and 400 mA for 40 min.

### 2.4. Sequencing and Nucleotide Sequence Analysis

The amplicons were purified using ExoSAP-IT^TM^ Express PCR Product Cleanup (Thermo Fisher Scientific, Waltham, MA, USA) following the manufacturer’s instructions and sequenced by a commercial sequencing service (Macrogen Europe, Amsterdam, The Netherlands). The obtained nucleotide sequences were analysed using the BioEdit software Version 7.2.5.0 (Tom Hall, Ibis Therapeutics, Carlsbad, CA, USA) and then aligned between them and against other homologous CIAV sequences available in the GenBank database (Table 1), using the Clustal W software [34].

### 2.5. VP1 Amino Acid Pattern Analysis

The VP1 amino acid sequences of the positive samples were aligned with homologous sequences of CIAV Del-Ros vaccine and Tunisian CIAV strains from the GenBank database to evaluate possible amino acid substitutions at specific positions previously identified as the sites of the most common polymorphisms [10,35,36].

### 2.6. Phylogenetic Analysis

Two phylogenetic trees, based on VP1 gene nucleotide sequences (1350 nucleotides in length) and complete genome sequencing (2181 nucleotides in length), respectively, were built using the Maximum Likelihood method by MEGA X [37]. Nodal supports were estimated with 1000 bootstrap replicates and considered reliable when equal to or greater than 70.

### 2.7. Recombination Analysis

Putative homologous recombination events in the Tunisian CIAV strains detected in this study were analysed using a dataset containing 105 additional CIAV genomes from GenBank (Table S1); we used multiple methods (RDP, Geneconv, Bootscan; Maxchi, Chimaera, Siscan and 3seq) implemented in the Recombination Detection Program 4 (RDP v. 4.97) [38]. The *p*-value was adjusted to 0.05. Only recombination events supported by no less than five independent detection methods were regarded as positive.

### 2.8. GenBank Accession Numbers

The full genome sequences obtained in this study were submitted to the GenBank database and are available under the following accession numbers: MZ666088–MZ666102.

## 3. Results

### 3.1. PCR CIAV Detection

Fifteen samples were positive for CIAV based on the VP1 nested PCR analysis (Figure S1). All positive samples were from the rural farm (Farm E/lot 7). The full genome sequence was obtained for all positive samples (Figure S2).

### 3.2. Nucleotide and Amino Acid Sequence Analysis

The VP1 sequence analysis showed that CIAV sequences obtained in this study were closely related to each other, with 99.5 and 100% mean identity at the nucleotide or amino acid level, respectively. They were similar to previously described Tunisian CIAV sequences, showing a VP1 mean identity of 98.6 and 99.7% at the nucleotide and the amino acid level, respectively. Interestingly, the VP1 amino acid identity between all new detected strains and TN 126/16 and TN 1328/09 previously reported Tunisian CIAV strains was 100%.

With regards to VP2 and VP3 genetic variability between the newly detected CIAV strains, mean identity was 99.8 and 99.9% at the nucleotide level and 100 and 99.6% at the amino acid level, respectively. Comparison between the new strains and those previously described in Tunisia showed VP2 and VP3 nucleotide mean identity levels of 99.5 and 99.9%, respectively. At the amino acid level, the mean identity was 99.3 and 99.7% for VP2 and VP3, respectively.

### 3.3. VP1 Amino Acid Pattern Analysis

All positive samples showed the same amino acid motif. The new strains showed aa substitutions in only two positions, namely 370S/T and 447T/S, when compared with CIAV VP1 TN200/11, TN1021/16 and TN1340/09 sequences previously detected in Tunisia. With respect to the Del-Ros reference vaccine strain, 10 major variable aa substitutions were detected in VP1 of the new Tunisian CIAV strains, namely 75V/I, 97M/L, 139K/Q, 144E/Q, 157V/M, 287S/T, 290A/P, 370G/T, 413S/A and 447G/S.

### 3.4. Phylogenetic Analysis of the CIAV Genome

**Figure 2:**
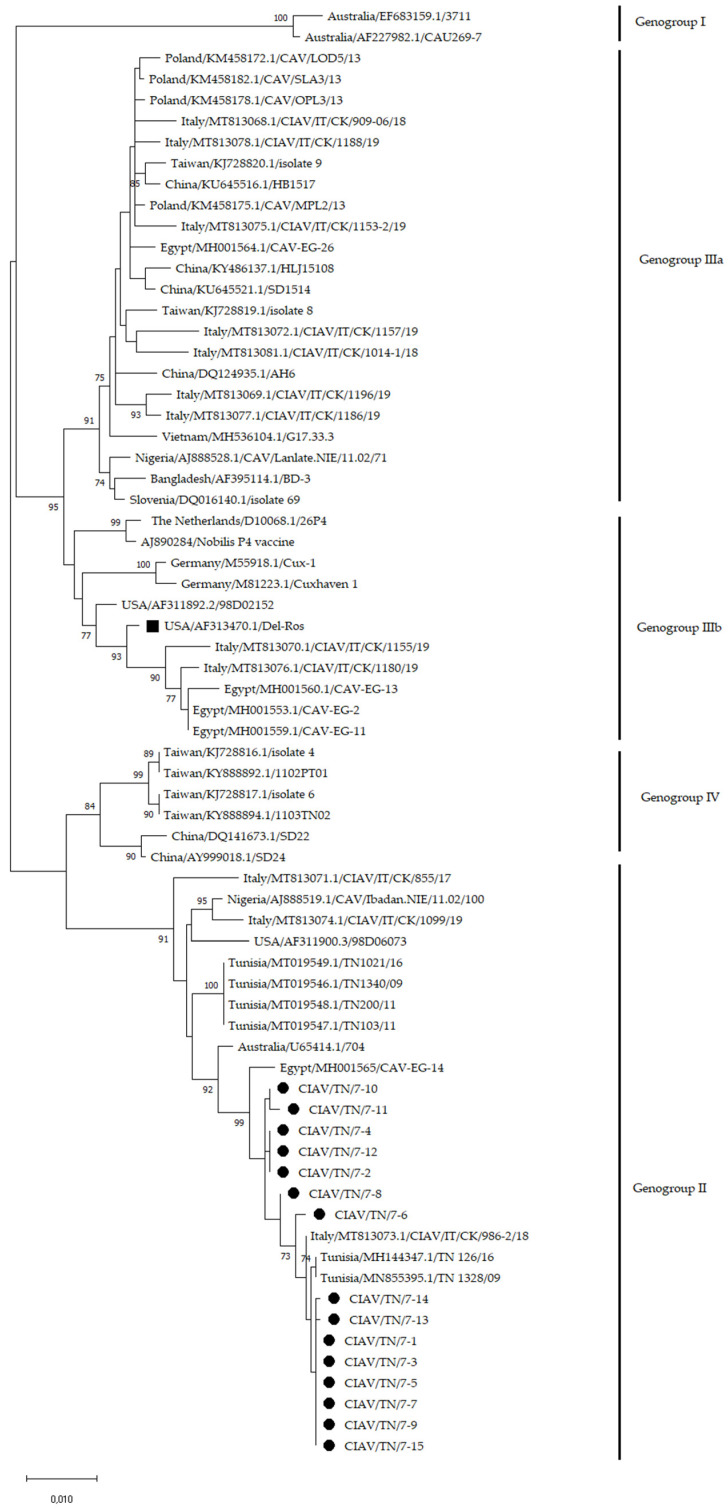
Phylogenetic tree based on the VP1 nucleotide sequence (1350 nt in length) of 15 Tunisian CIAV samples and CIAV strains from the GenBank database. New Tunisian CIAV sequences were marked with a black circle. Del-Ros vaccine strain was marked with a black square. Only bootstrap values ≥ 70 were reported. The tree was drawn to scale, with branch lengths in the same units as the evolutionary distances used to infer the phylogenetic tree.

The phylogenetic tree based on the VP1 CIAV nucleotide sequences is shown in Figure 2. Based on the nomenclature proposed by Ducatez [10] and Ou [14], all Tunisian samples analysed in the present study belonged to genogroup II and clustered with CIAV strains previously detected in Tunisia, Italy and Egypt. Similar results were obtained by the phylogenetic tree based on the CIAV complete genome sequences (Figure S3).

### 3.5. Recombination Analysis

No recombination event was detected in the samples examined.

## 4. Discussion

The poultry industry is of particular importance to many African countries in terms of meat supply, social plan (cheapest meat) and as a means of filling the deficits of other proteins [16]. Tunisian poultry farming has been experiencing remarkable development since the 1980s. Standard broilers are the main supplier of white meats. In 2020, the production of broiler meat reached nearly 132,000 tonnes, i.e., around 63% of the poultry meat, with an increase rate of 13.6% compared to 2012. Broilers are mainly produced by small-scale farmers, with 78% of farms having a capacity under 10,000 chickens per rotation, according to the last statistics (2015–2016) of the official Tunisian veterinary services and the Groupement interprofessionnel des produits avicoles et cunicoles [39]. However, the increase in the prices of raw materials is orienting production towards large integrated groups. In this context, backyard poultry consists of small flocks of different poultry species reared under traditional conditions. Free-range chicken farmers obtain day-old chicks from different farms from those that supply industrial farms or sometimes from informal circuits. Although backyard poultry represents an important socio-economic activity in rural areas of Tunisia, productivity in backyard flocks is often unsatisfactory because of serious health problems, malnutrition and poor management conditions.

With respect to the chicken infectious anemia virus circulation in the Tunisian poultry sector, the literature is limited to serological evidence in both breeders and broilers by Nsiri et al. [40]. The same authors reported the circulation of CIAV virulent strains in industrial poultry flocks, with many mutations detected in VP1 and VP2 genes. Phylogenetic similarities were identified with Indian strains [41]. To our knowledge, there are currently no scientific data available regarding CIAV circulation in Tunisian backyard poultry.

In the present study, DNAs extracted from cloacal swabs from 195 apparently healthy broiler chickens belonging to 13 lots from six Tunisian farms were examined for CIAV. Fifteen samples were detected to be positive for CIAV by using VP1 nested PCR analysis, and the full genome sequence was obtained for all positive samples. Nucleotide and amino acid sequence analyses showed a high degree of similarity between the sequences obtained, suggesting the circulation of a single CIAV strain in the lot confirmed positive. Phylogenetic analysis of CIAV VP1 nucleotide sequences or the complete genome showed that the sequences obtained in this study clustered with CIAV strains previously detected in Tunisia, Italy and Egypt, belonging to genogroup II. No recombination event was detected in the samples examined.

Analysis of amino acids at positions described previously as sites of the most common substitutions, including those useful for distinguishing CIAV strains [10,35,36], confirmed the lack of a relationship between CIAV Tunisian strains and the Del-Ros vaccine strain. The predicted aa at position 394 of VP1 of all Tunisian CIAV strains described in this study, shared with the previous reported CIAV Tunisian strains, was glutamine (Q). In this regard, Yamaguchi et al. [5] reported that viral pathogenicity could change from high to less pathogenic if the aa at position 394 in VP1 was substituted from glutamine to histidine. Glutamine at position 394 might therefore suggest high pathogenicity of the Tunisian CIAV strains. However, all newly sequenced CIAV Tunisian strains, as well as those previously reported in Tunisia, possessed Q at positions 139 and 144. Renshaw et al. [9] reported the association of VP1 139Q and/or 144Q with a decreased rate of growth or spread of CIAV in the cell culture. As the virulence-affecting amino acid substitutions in CIAV are not yet fully understood, additional in vivo studies should be conducted to properly evaluate the pathogenicity of Tunisian CIAV strains.

All CIAV-positive samples detected in this study were from a rural farm, whereas all sampled industrial farms were negative. The slaughter age of the chickens was 35–42 days for industrial chickens and 68 days for rural chickens. The difference in the age of slaughter depended on the genetic line raised on the rural farm, which is characterised by slow growth. All the sampled animals were apparently healthy. The investigated flocks did not report previous CIAV infections. Regarding the vaccination status of the breeders, six industrial lots were obtained from vaccinated breeders, four from unvaccinated breeders, whereas no information was available for two lots. Considering that the main measures to control CIAV spread include regular vaccination of breeder flocks combined with good poultry health and management practices [21], the negativity of all industrial farms sampled, both from vaccinated and unvaccinated breeders, suggested a rigorous application of biosecurity measures in their management. In particular, good control of the movements of vehicles and people, management of corpses and manure, control of disinfection and respect for crawl spaces were strictly applied, along with a homogeneous age of the farmed animals. In the future, these good management practices should be consolidated as usual practices, being the first barrier of defence to guarantee optimal profitability, minimising the losses linked to the appearance of health problems. Likewise, breeder vaccination programmes and serological evaluation of the vaccination efficacy in breeders should be implemented, involving all Tunisian breeder farms, by live attenuated vaccines administered a few weeks before the start of the lay period to prevent the vertical transmission of the virus and to promote the transfer of sufficient levels of maternal antibodies to chicks.

Regarding rural chickens, they were from an unvaccinated breeder stock. Our results suggested that they came into contact with the wild-type strain, although it is not possible to establish it temporally. The exposure of poultry to the CIAV strain could be linked to the frequent turnover of animals from informal circuits with the low hygienic-sanitary standard. Subclinical CIAV infection in rural chickens could be attributed to a contact with a hypo-virulent strain or a viral contact in the period of minor receptivity to infection (chickens older than two weeks of age). Furthermore, the hardiness of backyard chickens, such as in the case of the genetic line bred in the rural flock sampled, could make them more resistant to diseases and, therefore, a potential healthy reservoir of infections. This eventuality must be taken into particular consideration since potential healthy reservoirs may promote transmission of infection to industrial poultry farms, i.e., through the workers who raise birds at home. In this regard, the role of backyard chickens as a potential source of avian pathogens, including CIAV, for commercial chickens has repeatedly been emphasised [30,42], especially in densely poultry-populated areas, such as the one sampled in this study.

Our results suggest that regular screening of CIAV infection in backyard poultry flocks, particularly around industrial farms, should be provided for better control of CIAV infections.

## 5. Conclusions

Our study is limited in that only chicken farms located in a limited area of Tunisia and only one rural farm were examined. However, these preliminary results confirm the circulation of CIAV in the Tunisian poultry sector and suggest further investigations, especially on rural farms, considering the potential role of backyard chickens as a source of avian pathogens for intensive breeding.

## Figures and Tables

**Table 1:** CIAV sequences from GenBank included in this study.

CIAV Strain	GenBank Accession No.	Country	Genogroup
Del-Ros	AF313470	USA	IIIb
26P4	D10068	USA	IIIb
Nobilis P4 vaccine	AJ890284	The Netherlands	IIIb
Cux-1	M55918	Germany	IIIb
Cuxhaven 1	M81223	Germany	IIIb
CAU269-7	AF227982	Australia	I
3711	EF683159	Australia	I
BD-3	AF395114	Bangladesh	IIIa
CAV/Ibadan.NIE/11.02/100	AJ888519	Nigeria	II
CAV/Lanlate.NIE/11.02/71	AJ888528	Nigeria	IIIa
Isolate 69	DQ016140	Slovenia	IIIa
98D02152	AF311892	USA	IIIb
Isolate 4	KJ728816	Taiwan	IIIb
Isolate 6	KJ728817	Taiwan	IIIb
Isolate 8	KJ728819	Taiwan	IIIa
Isolate 9	KJ728820	Taiwan	IIIa
CAV-EG-2	MH001553	Egypt	IIIb
CAV-EG-11	MH001559	Egypt	IIIb
CAV-EG-13	MH001560	Egypt	IIIb
CAV-EG-14	MH001565	Egypt	II
CAV-EG-26	MH001564	Egypt	IIIa
CAV/LOD5/13	KM458172	Poland	IIIa
CAV/MPL2/13	KM458175	Poland	IIIa
CAV/OPL3/13	KM458178	Poland	IIIa
CAV/SLA3/13	KM458182	Poland	IIIa
G17.33.3	MH536104	Vietnam	IIIa
HB1517	KU645516	China	IIIa
AH6	DQ124935	China	IIIa
HLJ15108	KY486137	China	IIIa
SD1514	KU645521	China	IIIa
704	U65414	Australia	II
98D06073	AF311900	USA	II
1102PT01	KY888892	Taiwan	IV
1103TN02	KY888894	Taiwan	IV
SD22	DQ141673	China	IV
SD24	AY999018	China	IV
TN103/11	MT019547	Tunisia	II
TN 126/16	MH144347	Tunisia	II
TN200/11	MT019548	Tunisia	II
TN1021/16	MT019549	Tunisia	II
TN1340/09	MT019546	Tunisia	II
TN 1328/09	MN855395	Tunisia	II
CIAV/IT/CK/909-06/18	MT813068	Italy	IIIa
CIAV/IT/CK/1196/19	MT813069	Italy	IIIa
CIAV/IT/CK/1155/19	MT813070	Italy	IIIb
CIAV/IT/CK/855/17	MT813071	Italy	II
CIAV/IT/CK/1157/19	MT813072	Italy	IIIa
CIAV/IT/CK/986-2/18	MT813073	Italy	II
CIAV/IT/CK/1014-1/18	MT813081	Italy	IIIa
CIAV/IT/CK/1099/19	MT813074	Italy	II
CIAV/IT/CK/1153-2/19	MT813075	Italy	IIIa
CIAV/IT/CK/1180/19	MT813076	Italy	IIIb
CIAV/IT/CK/1186/19	MT813077	Italy	IIIa
CIAV/IT/CK/1188/19	MT813078	Italy	IIIa

## Data Availability

The sequences generated in this study are available in GenBank under Accession numbers MZ666088–MZ666102.

## Supplementary Materials

The following are available online at https://www.mdpi.com/article/10.3390/ani12010062/s1, Figure S1: Agarose gel of CIAV VP1 nested PCR product, Figure S2: Agarose gel of products obtained by three overlapping PCRs to amplify whole CIAV genome, Figure S3: Phylogenetic tree based on complete genome sequences (2181 bp) of fifteen Tunisian CIAV samples and CIAV reference strains from the GenBank database, Table S1: Reference strains from the GenBank database included in the recombination analysis.
