# Supplementary material for: Occurrence of Chicken Infectious Anemia Virus in Industrial and Backyard Tunisian Broilers: Preliminary Results
Source: Animals (Basel). 2021 Dec 28;12(1):62. doi: 10.3390/ani12010062 (PMC8749614; doi:10.3390/ani12010062)
Supplement: Supplementary file 1 [file animals-12-00062-s001.zip › animals-1471257-supplementary.pdf]

animals

Supplementary Material

Occurrence of Chicken Infectious Anemia Virus in Industrial and Backyard Tunisian Broilers: Preliminary Results

Antonietta Di Francesco [1,*], Giulia Quaglia [1], Daniela Salvatore [1], Sonia Sakhria [2], Elena Catelli [1], Ghaith Bessoussa [3], Khaled Kaboudi [4], Noureddine Ben Chehida [2] and Caterina Lupini [1]

1 Department of Veterinary Medical Sciences, University of Bologna, Ozzano dell'Emilia, 40064 Bologna, Italy; giulia.quaglia2@unibo.it (G.Q.); daniela.salvatore2@unibo.it (D.S.); elena.catelli@unibo.it (E.C.); caterina.lupini@unibo.it (C.L.)
2 Institute of Veterinary Research of Tunisia, University of Tunis El Manar, Tunis 1006, Tunisia; sakhrias@yahoo.fr (S.S.); nbenchehida@yahoo.fr (N.B.C.)
3 Commissariat Régional au Développement Agricole, Ben Arous 2063, Tunisia; ghaithbessoussa@hotmail.fr
4 National School of Veterinary Medicine of Sidi Thabet, University of Manouba, Sidi Thabet 2020, Tunisia; khaled.kaboudi@enmv.uma.tn
* Correspondence: antoniet.difrancesco@unibo.it

Citation: Di Francesco, A.; Quaglia, G.; Salvatore, D.; Sakhria, S.; Catelli, E.; Bessoussa, G.; Kaboudi, K.; Ben Chehida, N.; Lupini, C. Occurrence of Chicken Infectious Anemia Virus in Industrial and Backyard Tunisian Broilers: Preliminary Results. *Animals* **2022**, *12*, 62. https://doi.org/10.3390/ani12010062

Academic Editors: Seiya Yamayoshi and Antonio José Piantino Ferreira

Received: 2 November 2021
Accepted: 25 December 2021
Published: 28 december 2021

Publisher's Note: MDPI stays neutral with regard to jurisdictional claims in published maps and institutional affiliations.

Copyright: © 2021 by the authors. Submitted for possible open access publication under the terms and conditions of the Creative Commons Attribution (CC BY) license (https://creativecommons.org/licenses/by/4.0/).

Table S1. Reference strains from the GenBank database included in the recombination analysis.

CIAV strain	Country	GenBank Accession N°
Del-Ros	USA	AF313470
26P4	USA	D10068
Cux-1	Germany	M55918
Cuxhaven 1	Germany	M81223
CAU269-7	Australia	AF227982
3711	Australia	EF683159
BD-3	Bangladesh	AF395114
98D02152	USA	AF311892
Isolate 4	Taiwan	KJ728816
Isolate 6	Taiwan	KJ728817
Isolate 8	Taiwan	KJ728819
Isolate 7	Taiwan	KJ728818
Isolate 9	Taiwan	KJ728820
Isolate 18	Taiwan	KJ728827
Isolate 20	Taiwan	KJ728829
CAV-EG-2	Egypt	MH001553
CAV-EG-4	Egypt	MH001554
CAV-EG-6	Egypt	MH001555
CAV-EG-7	Egypt	MH001556
CAV-EG-10	Egypt	MH001557
CAV-EG-11	Egypt	MH001559
CAV-EG-13	Egypt	MH001560
CAV-EG-14	Egypt	MH001565
CAV-EG-15	Egypt	MH001568
CAV-EG-21	Egypt	MH001567
CAV-EG-23	Egypt	MH001562
CAV-EG-25	Egypt	MH001569
CAV-EG-26	Egypt	MH001564
CAV-EG-28	Egypt	MH001570
TR20	Japan	AB027470
N5	China	MK887167
G17.33.3	Vietnam	MH536104
AH6	China	DQ124935
HLJ15108	China	KY486137
LF4	China	AY839944
SD1514	China	KU645521
704	Australia	U65414
98D06073	USA	AF311900
1102PT01	Taiwan	KY888892
1103TN02	Taiwan	KY888894
SD22	China	DQ141673
SD24	China	AY999018
01-4201	USA	DQ991394
17JL0310	China	MK089241
3-1P60	Malaysia	AY040632
1504TW	Taiwan	MK358456
1535TW	Taiwan	MN299315
1637TW	Taiwan	MN299310
1852TW	Taiwan	MN299316

A2	Japan	AB031296
AH4	China	DQ124936
C14	China	EF176599
CAV/NAM/TANUVAS/09	India	KY053900
CAV-10	Argentina	KJ872513
CAV-18	Argentina	KJ872514
CAV-CA1-2015	Egypt	MG827098
CAV-SK4-2017	Egypt	MG827100
XH16	China	MK770259
N.A.	China	AF475908
CIAV89-69	South Korea	JF507715
CIAV-Mouse	China	KU645525
G17.33.3	Vietnam	MH536104
G6	Japan	AB119448
GD-102	China	KU050677
GD-K-12	China	KF224935
GX1904A	China	MN103402
GX1804	China	MK484615
HB1517	China	KU645516
HLJ14101	China	KY486136
HN1504	China	KU645512
JL14028	China	KY486148
LN1402	China	KU645511
LY-1	China	KX447636
SD1403	China	KU221054
SD1505	China	KU645523
SD1511	China	KU641015
SD1514	China	KU645521
SD1518	China	KU645522
SH11	China	DQ141670
SH16	China	DQ141671
HN9	China	DQ141672
TJBD33	China	AY843527
SDLY08	China	FJ172347
17SY0902	China	MK089243
SMSC-1	Malaysia	AF285882
SMSC-1P9WT	Malaysia	DQ217400
SMSC-1P123WT	Malaysia	DQ217401
TN103/11	Tunisia	MT019547
TN 126/16	Tunisia	MH144347
TN200/11	Tunisia	MT019548
TN1021/16	Tunisia	MT019549
TN1340/09	Tunisia	MT019546
TN 1328/09	Tunisia	MN855395
CIAV/IT/CK/909-06/18	Italy	MT813068
CIAV/IT/CK/1196/19	Italy	MT813069
CIAV/IT/CK/1155/19	Italy	MT813070
CIAV/IT/CK/855/17	Italy	MT813071
CIAV/IT/CK/1157/19	Italy	MT813072
CIAV/IT/CK/986-2/18	Italy	MT813073
CIAV/IT/CK/1014-1/18	Italy	MT813081

CIAV/IT/CK/1099/19	Italy	MT813074
CIAV/IT/CK/1153-2/19	Italy	MT813075
CIAV/IT/CK/1180/19	Italy	MT813076
CIAV/IT/CK/1186/19	Italy	MT813077
CIAV/IT/CK/1188/19	Italy	MT813078

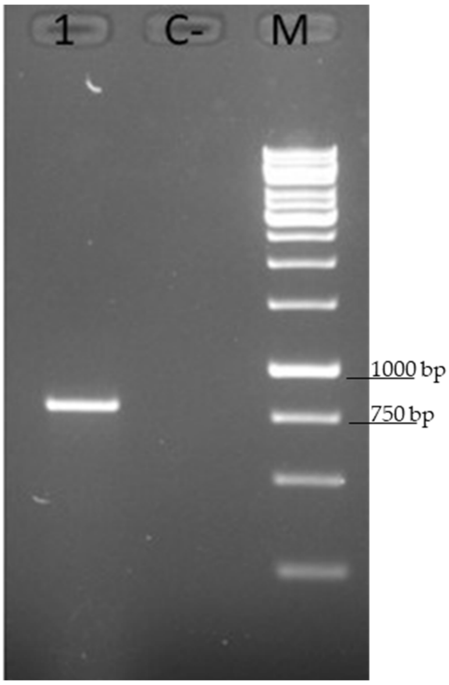

Figure S1. Agarose gel of CIAV VP1 nested PCR product. Lane 1: 790 bp purified product; Lane C-: negative control; Lane M: GeneRuler 1kb DNA ladder.

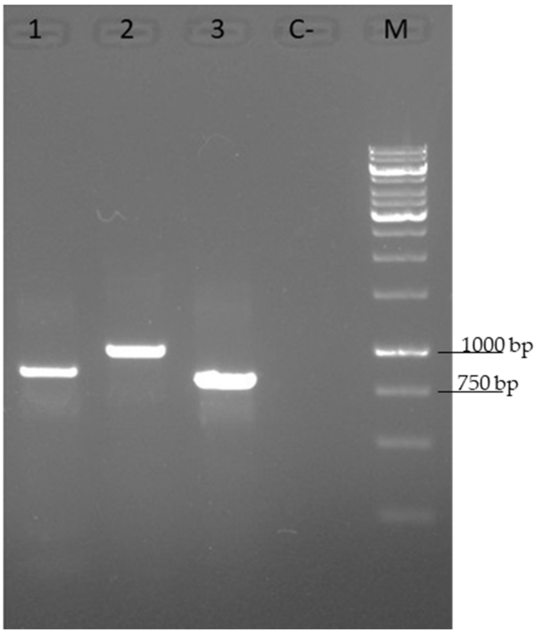

Figure S2. Agarose gel of products obtained by three overlapping PCRs to amplify whole CIAV genome. Lane 1: purified product of 843 bp; Lane 2: purified product of 989 bp; Lane 3: purified product of 802 bp; Lane C-: negative control; Lane M: GeneRuler 1 kb DNA ladder.

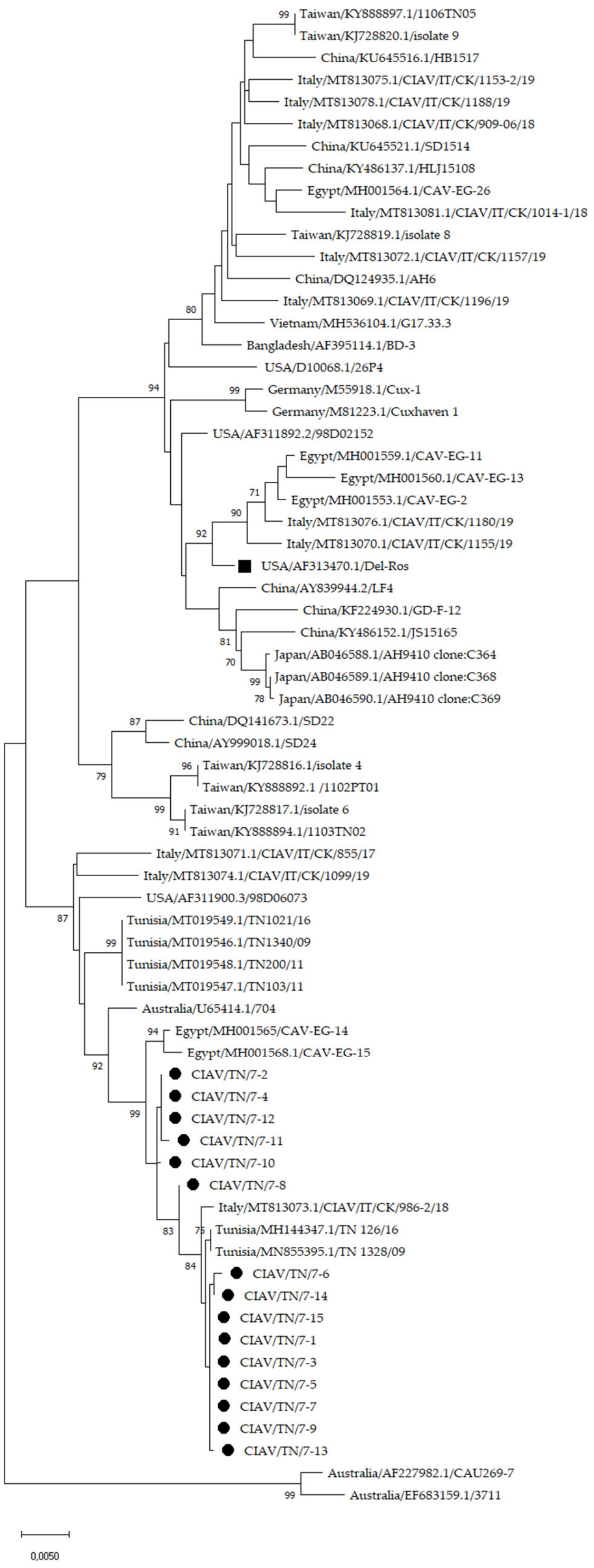

Figure S3. Phylogenetic tree based on complete genome sequences (2181 bp) of fifteen Tunisian CIAV samples and CIAV reference strains from the GenBank database. Tunisian CIAV sequences were marked with a black circle. Del-Ros Vaccine strain (GenBank accession No AF313470.1) was marked with a black square. Only bootstrap values ≥ 70 were reported. The tree was drawn to scale,

with branch lengths in the same units as those of the evolutionary distances used to infer the phylogenetic tree.
